# Comparative effects of moderate and high-intensity training on immune activation in myocarditis: a review of preclinical and clinical evidence

**DOI:** 10.3389/fphar.2025.1643056

**Published:** 2025-08-20

**Authors:** Bartolo Ferraro, Cristina Alejandra Buela Alvarado, Jonas Mink, Ludwig T. Weckbach

**Affiliations:** ^1^ Institute of Cardiovascular Physiology and Pathophysiology, Biomedical Center, Ludwig- Maximilian-University Munich, Munich, Germany; ^2^ Medizinische Klinik und Poliklinik I, Klinikum der Universität, Ludwig-Maximilians-University Munich, Munich, Germany; ^3^ Walter Brendel Centre of Experimental Medicine, Ludwig-Maximilians-University Munich, University Hospital, Munich, Germany; ^4^ DZHK, Partner Site Munich Heart Alliance, Munich, Germany

**Keywords:** myocarditis, immune cell activation/modulation, moderate intensity activity, high intensity activity, clinical studies, preclinical studies

## Abstract

Exercise intensity plays a critical role in influencing immune responses during myocarditis, a condition characterized by inflammation of the cardiac tissue. Based on preclinical and clinical evidence, this review examines how moderate *versus* high-intensity training affects immune activation in myocarditis. Studies involving animals suggest that moderate exercise may reduce inflammation and support immune regulation, while high-intensity training often exacerbates pro-inflammatory responses and worsens cardiac injury. Limited clinical data support these findings, indicating that moderate exercise could be safe or even beneficial in stable cases, whereas high-intensity exercise poses risks, particularly during active disease. Understanding these contrasting effects is essential for developing exercise recommendations for patients with myocarditis, balancing the need for recovery with immune safety. Further research is necessary to identify optimal exercise strategies for this vulnerable population.

## Introduction

Myocarditis is an inflammatory condition of the heart muscle (myocardium) triggered by a variety of factors, including viral infections, autoimmune reactions, and toxins (Brociek et al., 2023; Tschop et al., 2021). Persistent inflammation can lead to serious complications such as arrhythmias, chronic dilated cardiomyopathy, and ultimately heart failure (Sagar et al., 2012). While some cases of myocarditis can be severe, many present with mild symptoms or none at all (Bracamonte-Baran and Cihakova, 2017). Physical activity is widely recognized as essential for cardiovascular health (Valenzuela et al., 2023), but its role, particularly in cardiac diseases such as myocarditis, has raised questions about its dual potential as both a therapeutic and harmful factor. Current guidelines recommend abstinence from moderate to high physical activity for 3–6 months following a diagnosis of myocarditis (Pelliccia et al., 2020). However, emerging evidence suggests that aerobic exercise might support recovery during both the acute inflammation phase and the subsequent healing process (Bryde et al., 2023; Chen et al., 2024; Pelliccia et al., 2019). Diverging views on this topic arise from variations in study design, including differences in organisms used, timing, exercise intensity, and measurement criteria. Given the central role of the immune system in regulating inflammation in myocarditis (Forte et al., 2022; Scheffer and Latini, 2020), this review summarizes how moderate and high-intensity exercise affects the immune response in this disease, drawing on insights from both human studies and animal models, particularly mouse models. A literature search was conducted in PubMed from the earliest available date to May 2025. The search terms included: exercise, physical activity, training or sport, myocarditis or myocardial inflammation, immune system, animal models, and clinical study. Studies evaluating the influence of training on the immune system in general, or in the context of myocarditis, were included. Studies using special pharmacological agents or those exclusively focused on *in vitro* experiments were excluded.

## Myocarditis: a multifaceted inflammatory condition with a complex immune response

Due to the highly variable clinical presentation and the lack of specific non-invasive diagnostic methods, myocarditis often remains underdiagnosed (Bracamonte-Baran and Cihakova, 2017). Endomyocardial biopsy (EMB) is the gold standard for diagnosing myocarditis, as it is often the only method to identify the underlying etiology (Fung et al., 2016; Kindermann et al., 2012). However, because myocarditis typically displays patchy infiltration of immune cells, a single EMB may not provide sufficient sensitivity (Chow et al., 1989). To reduce sampling error, the analysis of at least five biopsies is recommended (Baughman, 2006). Like all invasive diagnostic methods, this carries potential risks, which may prevent doctors from obtaining the necessary samples (Tschop et al., 2021). For this reason, combining EMB with imaging techniques such as Magnetic Resonance Imaging (MRI) enhances the accuracy of disease identification (Tschop et al., 2021). The causes of myocarditis can be both infectious and non-infectious. Infectious agents include viruses, e.g., Coxsackievirus B3, Parvovirus B19, and Human Herpesvirus 6 (HHV6); bacteria such as *Staphylococcus* and *Streptococcus*; and protozoa such as *Toxoplasma gondii* and *Trypanosoma cruzi* (Vicenzetto et al., 2024). Non-infectious causes include toxins like amphetamines, cocaine, or immune checkpoint inhibitors (ICI), as well as autoimmune and systemic inflammatory conditions such as systemic lupus erythematosus and rheumatoid arthritis (Vicenzetto et al., 2024; Lampejo et al., 2021). More recently, SARS-CoV-2, the virus responsible for COVID-19, has been identified as a potential trigger for viral myocarditis, either through direct myocardial infection or as a result of immune-mediated injury (Siripanthong et al., 2020). In addition, rare cases of myocarditis have been reported following mRNA-based COVID-19 vaccination, particularly in young males, typically presenting within a few days after the second dose. These cases are usually mild and self-limiting but have raised awareness of post-vaccination inflammatory cardiac responses (Bozkurt et al., 2021; Oster et al., 2022). Histopathologically, myocarditis can be classified into several subtypes: acute or chronic lymphocytic myocarditis (most often of viral origin), giant cell myocarditis (believed to be linked to autoimmunity), sarcoidotic myocarditis, and eosinophilic myocarditis (Bracamonte-Baran and Cihakova, 2017). Experimental mouse models, particularly those using Coxsackievirus B3 to simulate viral myocarditis in humans and experimental autoimmune myocarditis (EAM) to mimic human giant cell myocarditis, are instrumental in studying the immune system´s role in the disease (Matsumori, 2023). The immune response in myocarditis progresses through distinct phases, each characterized by specific cellular and molecular mechanisms. In animal models, both viral and autoimmune myocarditis exhibit overlapping yet distinct immune-mediated pathophysiological processes. The acute phase typically lasts from day 0 to day 7 and occurs at both the systemic and myocardial tissue levels. During this phase, viral replication occurs (as seen in viral myocarditis models) alongside an innate immune response. Pattern recognition receptors (PRRs), such as Toll-like receptors (TLRs), play a crucial role in detecting viral RNA. This detection triggers the activation of pathways involving nuclear factor-kappa B (NF-κB) and type I interferons (IFN-α/β) (Alexopoulou et al., 2001). Consequently, inflammatory cytokines, including Interleukin-6 (IL-6), Tumor necrosis factor (TNF)-α, and Interleukin-1 beta (IL-1β), are upregulated. This leads to the infiltration of neutrophils, dendritic cells, monocytes, and macrophages (Tschop et al., 2021; Epelman et al., 2015). Mast cells are also among the first responders to viral infections in the heart (Higuchi et al., 2008). They degranulate within 6 h of infection and produce pro-inflammatory cytokines such as TNF, IL-1ß, and IL-4 (Tschop et al., 2021). In the study by Higuchi et al., it was observed that mast cell-deficient mice exhibited a higher survival rate, as well as reduced myocardial necrosis and less pronounced inflammatory cell infiltration. This finding confirms the significant role of mast cells in the pathogenesis of murine myocarditis (Higuchi et al., 2008). Neutrophils and monocytes contribute to the inflammatory cascade by secreting additional inflammatory cytokines (Swirski and Nahrendorf, 2018). Neutrophils are usually found in low numbers in healthy myocardium, but they are recruited to the site of infection and act as part of the first line of defence during myocarditis (Epelman et al., 2015). Neutrophils, by releasing neutrophil extracellular traps (NETs), drive myocardial inflammation, thus contributing to the pathogenesis of myocarditis (Weckbach et al., 2019). Monocytes are recruited to the cardiac tissue, and once they enter the myocardium, they start differentiating into macrophages (Epelman et al., 2015). Inflammatory macrophages secrete pro-inflammatory cytokines, such as TNF-α and IL-6, which contribute to tissue degradation and activate T cells. These events lead to the following subacute phase lasting 1–4 weeks, in which the adaptive immune response becomes the primary defence mechanism, with antigen-presenting cells (APCs) activating T-helper (Th) cells. In cases of viral myocarditis, Th1-mediated responses are the most prominent. These are characterized by the activation of interferon-gamma (IFN-γ) and cytotoxic T lymphocytes (CTLs), which can lead to apoptosis of cardiomyocytes. In models of autoimmune myocarditis, there is a shift towards an imbalance of Th17 and regulatory T cells (Tregs), which further exacerbates cardiac inflammation (Bracamonte-Baran and Cihakova, 2017). This phase is marked by peak myocardial injury, resulting from the interaction of pro-inflammatory and cytotoxic processes. The persistence of inflammation or immune system dysregulation can lead to myocardial fibrosis and heart failure during the final chronic phase, occurring after day 21. In certain models, ongoing immune activation results in the production of autoantibodies, further causing damage to the myocardium. Tregs play a crucial role in resolving inflammation, and a deficiency in these cells has been associated with the progression of chronic myocarditis (Shi et al., 2010). Additionally, eosinophils appear to be associated with the progression to dilated cardiomyopathy (DCM) (Swirski and Nahrendorf, 2018). Activating the innate immune response is vital for fighting myocarditis; however, an imbalance between pro-inflammatory and anti-inflammatory responses can result in an exaggerated or chronic inflammation (Huang et al., 2009). Despite the considerable body of existing knowledge, the immunological mechanisms underlying myocarditis are still not fully elucidated.

## Effects of moderate and high-intensity training on myocarditis

### Preclinical animal models

The effects of exercise on myocarditis have been studied using various animal models, particularly focusing on viral myocarditis, the most common type (Sagar et al., 2012). Many studies utilize the Coxsackievirus B3 (CVB3) myocarditis model (Favere et al., 2024; Gatmaitan et al., 1970; Kiel et al., 1989; Reyes et al., 1981; Ilback et al., 1989; Cabinian et al., 1990). In a recent study, mice underwent 8 weeks of moderate, forced treadmill training prior to receiving a single intraperitoneal injection of CVB3. The researchers observed that exercise influenced the inflammatory response in viral myocarditis (Favere et al., 2024). Specifically, the inflammatory infiltrates exhibited a higher cellular density and increased CD8^+^ T lymphocytes and macrophages (Favere et al., 2024). Both the trained and untrained groups infected with CVB3 developed myocardial fibrosis; however, the trained group showed a greater extent of interstitial fibrosis (Favere et al., 2024). This may explain why there is a tendency for ventricular arrhythmias in the group of infected animals exercising (Favere et al., 2024). A study conducted on mice infected with CVB3 demonstrated that exercising during the acute phase of the infection significantly increased the viral load in cardiac tissue and exacerbated myocardial inflammation and necrosis (Gatmaitan et al., 1970). Half of the mice in this study died during exercise due to congestive heart failure. Conversely, when swimming was introduced 9 days after the onset of the disease, both virulence and lethality only increased moderately (Gatmaitan et al., 1970). This suggests that exercise in the initial phase of infection worsens cardiac inflammation and tissue damage, ultimately deteriorating the overall health condition (Gatmaitan et al., 1970). Another study also used swimming as a method of forced exercise and found heightened virulence in the groups that exercised (Kiel et al., 1989). Specifically, this study revealed significantly increased viral titers on day 6 and day 9. These findings were linked to greater myocardial fibrosis, enlarged hearts, and an overall increase in mortality (Kiel et al., 1989). The CVB3 model is a reliable model for dilated cardiomyopathy, and exercise during the acute phase has also been shown to be the cause of larger atrial thrombi (Reyes et al., 1981). The presence of thrombi was linked to an increased risk of arrhythmias. The exercised group generally exhibited more significant issues related to left ventricular dilation, fibrotic scarring, and calcium deposits (Reyes et al., 1981). Exercise has also been linked to an increase in inflammatory cells, including CD8^+^ T cells, macrophages, and monocytes (Ilback et al., 1989). During myocarditis, maintaining a balanced immune response is crucial. This study demonstrated that exercise disrupts this balance. Specifically, there was an increase in pro-inflammatory cell types, such as CD8^+^ T cells, and M1 macrophages, while regulatory T cells (Treg) and M2 macrophages were slightly reduced (Ilback et al., 1989). Reducing T cell activity decreased the severity of myocarditis, even in physically active mice (Cabinian et al., 1990). Immunosuppression resulted in fewer negative effects on the heart due to exercise (Cabinian et al., 1990). In a different model of myocarditis associated with Chagas disease, it was observed that exercising while undergoing benznidazole treatment had harmful effects (Mendonca et al., 2019). Trained animals that did not receive treatment exhibited higher levels of inflammation and oxidative stress, along with increased pro-inflammatory cytokines such as IL-6 and TNF-α (Mendonca et al., 2019). However, treatment effectively reduced markers of inflammation, oxidative stress, and overall myocardial damage, demonstrating its cardioprotective effects (Mendonca et al., 2019). Notably, combining exercise with treatment diminished these positive outcomes. These findings suggest that exercise may exacerbate inflammation and oxidative stress to a degree that counteracts the benefits of benznidazole treatment (Mendonca et al., 2019). One recent study, using a chronic Chagas cardiomyopathy (CCC) model in hamsters, found that 8 weeks of treadmill aerobic physical training (APT) reduced inflammation and fibrosis. APT was performed at moderate intensity and improved left ventricular remodeling and cardiac dysfunction. Significant differences were observed in the degree of myocardial inflammation, but not in the extent of total fibrosis. Damasceno et al. also reported that the sedentary group exhibited progressively worsening perfusion defects throughout the experiment (Damasceno et al., 2024). Finally, another recent study using the same disease model, but in C57BL/6 mice, showed that 5 weeks of aerobic training reduced fibrosis in both cardiac and skeletal muscle in diseased animals, although the number of infiltrating inflammatory cells remained unchanged. Moreover, trained mice exhibited reduced severity of cardiac arrhythmias and lower expression of pro-inflammatory and pro-fibrotic genes in the heart (Improta-Caria et al., 2025).

Most of these studies, summarized in Table 1, point towards exercise being detrimental to the course of myocarditis. However, it is crucial to emphasize that the intensity of physical training significantly influences whether physical activity creates a more pro-inflammatory environment. Another important limitation is that nearly all existing animal studies on myocarditis have been conducted in male animals. These potential sex-based immune differences should also be taken into account before drawing any general conclusions.

**TABLE 1:** Overview of preclinical studies investigating the effects of moderate- and high-intensity exercise on immune responses, viral load, myocardial inflammation, and fibrosis in myocarditis models. The table highlights the timing and type of exercise, associated immune and pathological outcomes, and mortality.

	Mortality	Macroscopical findings	Microscopical findings	Arrhythmia	Virulence
Gatmaitan et al. (1970)	↑ 50% in the exercised group (day 1) and 13.9% (day 9) vs. 5.5%	↑ larger hearts in the exercised group	↑ Inflammatory lesions	-	↑
Favere et al. (2024)	↓ 11% in the exercised group vs. 27% in the sedentary group 0% in the pretrained group vs. 30% in the presedentary group	↑ cardiac interstitial fibrosis	↑ cellular density of inflammatory infiltrates, ↑ CD8^+^ T cells, and macrophages in the exercised group	↑ arrhythmia burden	-
Kiel et al. (1989)	↑ 20%–40% in the exercised group vs. 0%–10%	↑ heart:body weight ratios ↑ myocardial fibrosis	-	-	↑
Reyes et al. (1981)	↑ 45% in the exercised group vs. 13,2%	↑ larger hearts ↑ diffuse areas of epicardial scar ↑ heart:bodyweight ratios	↑ microscopic calcification	↑ atrial thrombi were found, suggesting more atrial arrhythmias	-
Ilback et al. (1989)	no alteration in disease-related lethality	↑ inflammatory and necrotic lesions (exercise starting 48 h after myocarditis induction)	↑ CD8^+^ T cells ↓ MHC-II expressing cells	-	-
Cabinian et al. (1990)	↑ 52% in the exercised group vs. 0% in the acute phase; overall: 72% in the exercised group vs. 4%	↑ heart: bodyweight ratios	↑ myocardial inflammation and necrosis	-	↑
Damasceno et al. (2024)	overall: 16% mortality rate	-	↓ skeletal muscle inflammation in the infected trained group	-	-
Improta-Caria et al. (2025)	-	-	↑ cardiac fibrosis in SED group ↑ skeletal muscle fibrosis in SED group Similar amount of inflammatory cells in heart and skeletal muscle	Exercise mitigates severity of arrhythmia	-

### Current standards for resuming exercise and clinical evaluation post-myocarditis

As described in the introduction, following a diagnosis of myocarditis, current guidelines recommend a period of physical activity restriction for all individuals, regardless of age or athletic status (Pelliccia et al., 2019). This is due to the heightened risk of arrhythmias, heart failure, or sudden cardiac death that can occur with exercise during active inflammation. Typically, moderate to high-intensity physical activity, including recreational exercise and competitive sports, should be avoided for at least 3–6 months. During this time, the focus is on cardiac recovery and careful monitoring. Before returning to physical activity, a comprehensive evaluation is required. This includes assessment of symptoms, cardiac biomarkers (such as troponin and inflammatory markers), and cardiac function through echocardiography (Bryde et al., 2023). Additional testing, like 24-h Holter monitoring and exercise stress testing, helps identify any residual electrical or functional abnormalities (Bryde et al., 2023). Cardiac Magnetic Resonance Imaging is commonly used to detect prolonged myocardial inflammation or fibrosis; the presence of late gadolinium enhancement (LGE) may indicate a higher risk and warrant further caution (Bryde et al., 2023). LGE relies on the use of a gadolinium-based contrast agent, which distributes in the extracellular space. In damaged myocardial tissue, the extracellular volume is increased, allowing more gadolinium to accumulate and wash out more slowly compared to normal myocardium. Areas of damage or scarring appear hyperintense in MRI (Aquaro et al., 2023). Once the heart has returned to normal function, inflammation has resolved, and there are no concerning findings on imaging or rhythm monitoring, a gradual and supervised return to physical activity can be considered. This approach applies broadly, from sedentary individuals to elite athletes and across all age groups, to ensure safety and prevent long-term complications (Hurwitz and Issa, 2020; Terry et al., 2024; Maisch, 2015; Van Name et al., 2024). It remains unclear whether this approach is fully scientifically justified. For example, Halle et al. recommended that athletes with uncomplicated myocarditis can resume physical activity as early as 1 month after the acute phase (Halle et al., 2021). Depending on the clinical appearance of the patient, a few studies recommend performing the investigation earlier and starting with moderate activity before 3 months post-inflammation (Schmidt et al., 2022). Overall, these studies provide insight into how individualized decisions should be made, especially in complex or borderline cases, and underscore the importance of a cautious, evidence-based approach to resuming exercise after myocarditis.

### Human studies: sport - curse or blessing?

Sport is recognized as a crucial protective factor against many lifestyle diseases. Sedentary behaviour combined with the consumption of high-calorie foods can lead to increased fat levels and various lifestyle diseases, such as respiratory and cardiovascular diseases (Silveira et al., 2022). On the other hand, some studies indicate that high-intensity exercise may increase the risk of contracting infections due to a temporary suppression of the immune system. This phenomenon might be explained by a reduction in natural killer (NK) cells and neutrophils that is observed after intense workouts (Dick and Diehl, 2014). Additionally, the downregulation of lymphocyte proliferation, monocyte toll-like receptors, and other important immune functions could also contribute to this risk (Gleeson, 1985). Furthermore, excessive endurance training can elevate levels of adrenaline, cortisol, prolactin, and growth hormone, as well as ROS due to a higher electron flow in the mitochondria, which may harm cellular immunity (Dick and Diehl, 2014; Harris, 2011; Nieman and Wentz, 2019; Chevion et al., 2003). Athletes may experience increased susceptibility to infections after events like marathons, and this is influenced by multiple factors. It is not solely determined by how exercise affects the immune system (Simpson et al., 2020). Therefore, accurately distinguishing the specific impact of exercise and its effects on immunity can be quite challenging. Current research distinguishes between moderate and high-intensity interval training (HIIT), highlighting their different effects on the immune system and their varying suitability for specific applications and goals. Regarding the vascular system, HIIT is significantly more time-efficient than moderate-intensity training in enhancing vascular function (Ramos et al., 2015). Additionally, the positive effects of HIIT have been observed in breast cancer survivors, contributing to improvements in cardiovascular fitness, cardiac regulation, and the sympathetic nervous system (Toohey et al., 2020). HIIT is often praised for its efficiency in achieving similar improvements in aerobic capacity (VO_2peak_) and reducing systemic inflammation markers (Bartlett et al., 2017). However, it is important to note that high-intensity exercise can also contribute to increased inflammatory responses, especially concerning upper respiratory tract infections (URTIs) (Terry et al., 2024).

How does physical training, in general, impact the immune system? Table 2 summarizes several studies exploring the effects of different types of training on the human body. Although there are exceptions, most studies in the literature differentiate between moderate- and high-intensity training.

**TABLE 2:** Overview of clinical studies evaluating how different intensities of physical exercise affect the immune system in humans. The table highlights effects on immune cell populations, systemic inflammation, susceptibility to infections, and exercise-related outcomes in various populations.

	Exercise in general (unclear specification in the study)	Influence of moderate-intensity training	Influence of high-intensity training
Dick and Diehl, (2014)	-	↑ IgA levels ↑ muscle-derived IL-6 => ↑ type 2 T-cell cytokine production => ↓ tissue damage through the immune system ↓ TNF-α	↓ T-cell proliferation ↑ risk of viral infection ↓ 50%–65% reduced IgA levels ↓ NK cells, neutrophil phagocytic function ↑ stress hormones (adrenaline, cortisol, prolactin, and growth hormone)
Gleeson, (1985)	↓ toll-like receptor expression ↓ neutrophil respiratory burst ↓ lymphocyte proliferation (temporary) ↓ monocyte antigen presentation (∼3–24 h post-exercise) ↓ immune function (in intensified training) ↑ ROS, TNF-α, CRP ↑ gut permeability ↑ anti-inflammatory cytokines (IL-6, IL-10, IL-1 receptor antagonist) ↑ stress hormones (adrenaline, cortisol, prolactin and growth hormone) ↑ type 2 T cells (less tissue damage/inflammation)		
Harris, (2011)	-	↑ Neutrophil/NK cells ↑ salivary IgA ↓ URTI incidence (∼20–30%)	↓ NK cells ↓ secretory IgA ↓ Lymphocyte/neutrophil cells/B cell function ↑ stress hormones (adrenaline, cortisol, prolactin and growth hormone) ↓ serum IgG/IgE
Simpson, R.J. et al. (2020)	↑ steroid hormone production, adrenaline exposure => can influence ↑ may enhance immune competency	↑ exchange of immune cells between circulation and peripheral lymphoid tissues	↓ Mucosal response (innate and acquired immune parameters) ↓ NK cell/neutrophil function ↓ lymphocyte function ↓ salivary IgA ↓ MHC II expression in macrophages ↓ cytotoxic T cells (over years of intensified training) ↓ immune function ↑ Leukocyte frequency (↑ in beginning, then ↓) ↓ cytokine production ↑ identification and eradication of tumor cells ↑ reverse T cell immunosenescence (apoptosis of old T cells)
Ramos et al. (2015)	↑ vascular function ↓ SBP (rather in HIIT, unclear) ↑ insulin sensitivity no change in CRP ↓ body fat (in HIIT slightly more than MICT)	-	↑ HIIT is more potent than MICT ↑ FMD (significantly greater following HIIT than MICT) ↑ cardiorespiratory fitness – VO2 (improved to a greater extent in HIIT) ↑ positive influence on lipid profile ↓ ROS (trend)
Bartlett et al. (2017)	↑ VO2 peak (both groups) ↑ neutrophil phagocytosis ↑ monocyte phagocytosis ↑ monocyte burst ↓ cortisol (trend) no association with reduction of systemic inflammation	↓ Leptin (significant) ↓ body fat	↑ Neutrophil oxidative burst (higher than MICT)
Maisch, (2015)	↑ Beneficial effect on: heart, vascular endothelium, skeletal muscle, and the central nervous system	↓ ROS	↑ ROS (possibly) ↑ NT-pro-BNP, Troponin
Chastin et al. (2021)	No effect on the overall lower white blood cell count ↓ neutrophil count ↓ pooled lower lymphocyte count ↑ pooled higher T4+ cell count	-	-
Dixit et al. (2023)	↓ neutrophil chemotaxis ↑ release of endothelium-derived NO ↑ IL4,6,16,15 (muscle discharges)	↑ neutrophil count ↑ lymphocyte counts	No effect on lymphocytes ↑ leucocyte count
Meyer-Lindemann et al. (2023)		Acute ↑ moderate leucocyte count (lymphocytosis and granulocytosis) ↑activation of neutrophils Chronic ↑improved immunosurveillance (better migration of NK-cells/T-cells) ↑Treg cells ⇨ anti-inflammatory	Acute ↑ leucocyte count ↑ monocyte count Reduction of TLRs Creation of an anti-inflammatory environment Chronic ↑TH2 cell count ↑Treg cells ⇨ anti-inflammatory
Nieman and Wentz, (2019)	Less than 60 min ↑ recirculation of immunoglobulins ↑ anti-inflammatory cytokines ↑ neutrophile counts ↑ NK cells ↑ cytotoxic T cells, immature B cells acute bouts: ↑ mobilization of NK cells and CD8^+^ T lymphocytes ↑ stimulation of the exchange of leukocytes between the circulation and tissues	↓ Stress hormones (limited) ↑ IgA salivary increases ↑ T cell function ↑ NK activity ↑ macrophage function ↑ granulocyte function ↑ cytokines (moderate)	↓ immune function ↑ inflammation ↑ ROS ↑ muscle damage ↑ NK cell count ↑ neutrophil function ↑ T-/B-cell function ↓ salivary IgA output ↓ MHC II expression in macrophages (also during recovery) ↓ NK activity ↓ macrophage function ↑ stress hormones increase ↑ cytokines
Petersen and Pedersen, (1985)	↑ IL-6 (produced by muscle fibers) => ↑ IL-1ra and IL-10 ↓ cytokine TNF-α ↑ lipid turnover, stimulating lipolysis, and fat oxidation ↓ TNF alpha-α	-	-
Friman and Ilback, (1998)	-	↑ positive impact on febrile respiratory infections	↓ susceptibility to infection (prior to infection) ↑ lethality (during infection)
Chevion et al. (2003)	-	-	↑ Oxygen uptake increases ↑ ROS augments (electron leakage in mitochondria)

Moderate intensity exercise refers to physical activity that causes a noticeable increase in heart rate and breathing. It typically corresponds to 40%–59% of an individual’s maximal oxygen uptake (VO_2_max), which is the maximum rate at which an individual can take in, transport, and use oxygen during intense exercise; or 50%–70% of maximum heart rate (HRmax), performed for at least 150 min per week, typically spread across at least 3–5 days (Harris, 2011; Garber et al., 2011). Constant moderate activity is associated with fewer community-acquired infections and strengthens the body’s first line of defence (Chastin et al., 2021). Regular moderate-intensity exercise boosts immune function, enhances the body’s antioxidant defences, and reduces oxidative stress. This leads to more efficient energy production and a lower risk of developing inflammatory diseases (Scheffer and Latini, 2020). It also tends to increase serum and salivary IgA concentrations, which may partly explain its protective effect, as these immunoglobulins are key components of the first line of defence (Dick and Diehl, 2014; Harris, 2011; Nieman and Wentz, 2019; Nieman et al., 1998). Higher levels of IgA are associated with improved mucosal immunity, which plays a critical role in protecting against respiratory infections. The immune system may also benefit from reduced levels of stress hormones (e.g., adrenaline, cortisol, and growth hormone) and decreased circulating ROS (Maisch, 2015; Nieman and Wentz, 2019; Bartlett et al., 2017). Furthermore, increases in lymphocyte, neutrophil, and NK cell counts have been observed following moderate exercise (Dick and Diehl, 2014; Harris, 2011; Nieman and Wentz, 2019; Dixit et al., 2023; Meyer-Lindemann et al., 2023). These cellular changes are supported by improved trafficking of immune cells between the bloodstream and peripheral lymphoid tissues, leading to a more effective immune response (Simpson et al., 2020; Meyer-Lindemann et al., 2023).

Interestingly, in the context of ongoing inflammation, moderate-intensity exercise appears to reduce excessive immune activation (Scheffer et al., 2019). One possible explanation is the muscle-derived release of IL-6 during exercise. IL-6 acts as both a pro- and anti-inflammatory cytokine; in the later, chronic phase of inflammation, its anti-inflammatory effects tend to dominate (Hunter and Jones, 2015). This may be due, in part, to its ability to suppress TNF-α (Dick and Diehl, 2014; Petersen and Pedersen, 1985). Moderate exercise has also been shown to benefit cardiac patients, for example, by improving cardiovascular fitness (Maisch, 2015). In cardiorespiratory diseases, there is evidence that moderate training exerts anti-inflammatory effects, which are essential for resolving persistent inflammation (Dixit et al., 2023). Similarly, in cancer patients, a reduction in pro-inflammatory markers has been observed with this type of physical activity (Khosravi et al., 2019).

High-intensity training, defined as exercise performed at 70%–80% of maximum heart rate for 5–60 min, and prolonged exercise sessions (typically exceeding 60 min), tend to exert negative effects on the immune system, particularly in the short term (Dick and Diehl, 2014; Harris, 2011; Friman and Ilback, 1998). Such activity decreases T-cell proliferation, thereby increasing susceptibility to viral infections and leading to general immunosuppression (Dick and Diehl, 2014). In addition, this type of training elevates stress levels. Stress hormones such as adrenaline and cortisol are significantly increased during strenuous exercise (Dick and Diehl, 2014; Harris, 2011). Some studies also report an increase in ROS during high-intensity training (Supruniuk et al., 2023). For instance, ROS are generated in the mitochondria of muscle cells during and after exercise (Chevion et al., 2003). Other sources include the activation of neutrophils, migrating into the muscle to repair activity-induced damage, and flow-induced ROS production in the endothelium (Chevion et al., 2003). Ultimately, caution must be taken not to exceed safe intensity levels. Excessive training can lead to myocardial fibrosis due to repeated micro-injuries, thereby increasing the risk of sudden cardiac death (SCD) (van de Schoor et al., 2016). This risk is particularly elevated in the presence of acute myocarditis. During training sessions of very high intensity, such as marathon running, even severe organ damage can occur, affecting the liver, intestines, and kidneys. Increased blood flow to the working muscles may lead to hypoperfusion of internal organs, potentially resulting in organ damage and triggering systemic inflammatory responses (Suzuki et al., 2020). However, strenuous exercise is not solely associated with negative effects on the immune system. Recent research indicates that HIIT can lead to significant improvements in cardiovascular fitness, metabolic health, and exercise capacity. Notably, these benefits are often achieved with a lower total exercise volume compared to moderate-intensity training (Soylu et al., 2021; Atakan et al., 2021). Interestingly, in a study examining the effects of high-intensity training in patients with hypertrophic cardiomyopathy, where participants were assigned to either moderate- or high-intensity training, no significant differences were observed between the groups in terms of fitness gains and cardiovascular response (MacNamara et al., 2023).

We can conclude that not all forms of exercise are the same. Different training intensities may be appropriate for different objectives and populations.

## Conclusion

The impact of physical activity on immune activation during myocarditis is a nuanced area that reveals both potential benefits and risks. Overall, current evidence indicates that different types of physical activity can have significant effects, both positive and negative, on cardiovascular and immune health, depending critically on the individual’s health status. In the setting of myocarditis, emerging data suggest that moderate activity may offer short-term support to immune function, potentially aiding in recovery. In contrast, high-intensity exercise can transiently suppress immune function and may exacerbate myocardial inflammation, particularly when performed during ongoing inflammatory states. Regular, moderate physical activity has been shown to exert anti-inflammatory effects, helping to regulate immune responses and potentially supporting healing. However, intense or excessive exercise could lead to heightened immune activation and worsen the condition, so it is essential to avoid strenuous activity until inflammation has subsided. These insights, while promising, do not currently justify deviation from guideline-based recommendations. The evidence remains insufficient to formally incorporate exercise-based therapies into the clinical management of myocarditis. Future research should focus on long-term studies that examine the sustained impact of structured exercise programs in post-myocarditis patients, aiming to establish safe and individualized exercise prescriptions. Such efforts should include investigations into the underlying molecular mechanisms, specifically involving cytokines, immune cell dynamics, and inflammatory markers, that mediate exercise-related immune modulation in myocarditis. Personalized exercise approaches based on immune profiling could ultimately provide a new therapeutic avenue in cardiovascular care.

For the general, healthy population, there is consistent evidence supporting moderate-intensity training as beneficial for preserving immune function and preventing cardiometabolic diseases. However, translating this into universal exercise guidelines remains a major public health challenge, especially considering the wide interindividual variability in disease severity, age, sex, comorbidities, baseline fitness, and even the etiology of myocarditis. While tailored exercise programs would be ideal, they are not yet realistic for broader application. What remains clear is that moderate exercise holds significant potential for supporting immune balance and cardiac recovery, but its integration into myocarditis management must proceed cautiously, guided by robust human data. Gaining a deeper understanding of how physical activity, particularly at moderate intensity, influences immune responses during myocarditis will be critical for ensuring patients receive the benefits of exercise without incurring harm.
